# Role of interleukin-3 as a prognostic marker in septic patients

**DOI:** 10.5935/0103-507X.20180064

**Published:** 2018

**Authors:** Isabela Nascimento Borges, Carolina Braga Resende, Érica Leandro Marciano Vieira, José Luiz Padilha da Silva, Marcus Vinícius Melo de Andrade, Andrea Jerusa de Souza, Eurípedes Badaró, Rafael Mourão Carneiro, Antônio Lúcio Teixeira Jr., Vandack Nobre

**Affiliations:** 1 Programa de Pós-graduação em Ciências da Saúde, Infectologia e Medicina Tropical, Departamento de Clínica Médica, Hospital das Clínicas, Faculdade de Medicina, Universidade Federal de Minas Gerais - Belo Horizonte (MG), Brasil.; 2 Laboratório Interdisciplinar de Investigação Médica, Faculdade de Medicina, Universidade Federal de Minas Gerais - Belo Horizonte (MG), Brasil.; 3 Departamento de Estatística, Universidade Federal de Santa Maria - Santa Maria (RS), Brasil.; 4 Programa de Pós-graduação em Ciências da Saúde: Saúde do Adulto, Departamento de Clínica Médica, Hospital das Clínicas, Faculdade de Medicina, Universidade Federal de Minas Gerais - Belo Horizonte (MG), Brasil.

**Keywords:** Interleukin-3, Sepsis, Septic shock, Biomarkers

## Abstract

**Objective:**

To evaluate the accuracy of IL-3 to predict the outcome of septic patients.

**Methods:**

Prospective cohort study with adult patients in an intensive care unit with sepsis or septic shock diagnosed within the previous 48 hours. Circulating IL-3 levels were measured upon inclusion (day 1) and on days 3 and 7. The primary outcome was hospital mortality.

**Results:**

One hundred and twenty patients were included. Serum levels of IL-3 on day 1 were significantly higher among patients who died than among patients who survived the hospital stay (91.2pg/mL *versus* 36pg/mL, p = 0.024). In a Cox survival model considering the IL-3 levels at inclusion, age and sequential SOFA, IL-3 values remained independently associated with mortality (HR 1.032; 95%CI 1.010 - 1.055; p = 0.005). An receiver operating characteristic curve was built to further investigate the accuracy of IL-3, with an area under the curve of 0.62 (95%CI 0.51 - 0.73; p = 0.024) for hospital mortality. A cutoff initial IL-3 value above 127.5pg/mL was associated with hospital mortality (OR 2.97; 95%CI: 1.27 - 6.97; p = 0.0019) but with a low performance (82% for specificity, 39% for sensibility, 53% for the positive predictive value, 72% for the negative predictive value, 0.73 for the negative likelihood and 2.16 for the positive likelihood ratio).

**Conclusion:**

Higher levels of IL-3 are shown to be independently associated with hospital mortality in septic patients but with poor clinical performance.

## INTRODUCTION

Sepsis represents an important public health issue, being present in 30% of patients undergoing intensive care.^(1)^ Despite recent therapeutic advances, sepsis mortality rates remain high, reaching 50% in more severe cases.^(1,2)^ Among survivors, the high rates of hospital readmission and functional decline further increase the social and economic burden of this syndrome.^(3,4)^

Multiple aspects of the pathophysiology of sepsis have yet to be elucidated.^(5)^ The role of pro-inflammatory cytokines and the exacerbated inflammatory response in tissue injury and death have been well established in recent years.^(5-7)^ However, clinical trials carried out to evaluate anti-inflammatory therapies have yielded predominantly unfavorable results.^(8)^

For this reason, new steps in the inflammatory cascade are being studied. Weber et al. showed the role of interleukin-3 (IL-3) in emergency myelopoiesis after sepsis induction in a murine model.^(9)^ The cytokine was revealed to be responsible for the mobilization and proliferation of myeloid cells, instigating the excessive release of pro-inflammatory cytokines and, consequently, systemic inflammation, organ dysfunction and death. In that study, the authors found an association between higher IL-3 levels and mortality in two small cohorts of sepsis patients.^(9)^

The objective of this study was to evaluate the accuracy of IL-3 to predict the outcome of septic patients, considering in-hospital mortality as the primary outcome. As secondary endpoints we evaluated 28-day mortality and intensive care mortality and measured the levels of other traditional biomarkers of sepsis.

## METHODS

Patients were participants in a prospective cohort of septic patients in an intensive care unit (ICU) with 18 beds in the *Hospital das Clínicas* at the *Universidade Federal de Minas Gerais* (UFMG), Brazil.

Given that the conduction of this study preceded the publication of the new definitions proposed by the Third International Consensus Definitions for Sepsis and Septic Shock,^(10)^ the sepsis definitions published in 1992 and revised in 2002^(11,12)^ were adopted. As such, every adult patient (≥ 18 years old) admitted with confirmed or suspected severe sepsis (infection plus systemic inflammatory response syndrome - SIRS plus at least one infection-related new organ dysfunction) or septic shock (infection plus SIRS plus hypotension with need of vasopressors) diagnosed in the previous 48 hours was considered for inclusion. The exclusion criteria were: (1) use of therapeutic antibiotic therapy against the current infectious process for longer than 48 hours; (2) patients undergoing palliative care only; (3) patients expected to be deceased in the next 24 hours; (4) severely immunosuppressed patients (HIV infection with CD4+ lymphocyte levels < 200/mm^3^; severe neutropenia < 500/mL; post-solid organ or bone marrow transplant patients; patients undergoing steroid therapy with immunosuppressive dose (dose equivalent to 10mg of prednisone for 30 days or more or 40mg of prednisone for 10 days or more); patients using chemotherapeutic agents in the last 28 days); and (5) polytraumatized patients or those submitted to major surgeries in the last five days (except surgery for control of the infectious focus).

The study was approved by the Ethics Committee of the UFMG, approval protocol number 0319.0.203.000-11, and all inclusions required signing of an informed consent form by the patient or surrogate.

Patients were assessed for potential inclusion at the time of admission in the ICU or immediately after the diagnosis of sepsis, in case they were already at the ICU. The clinical data were collected prospectively, and the following variables were recorded: age; sex; comorbidities; type and focus of infection; microbiological data; serum arterial lactate, C-reactive protein (CRP), creatinine, urea, hemoglobin, hematocrit, platelets, leukocyte, bilirubin, arterial pH, prothrombin time and aPTT; Acute Physiology and Chronic Health Evaluation (APACHE II)^(13)^ and Sepsis Organ Failure Assessment (SOFA) severity scores;^(14,15)^ duration and class of antibiotic therapy; durations of hospital stay and stay under intensive care; and all-cause mortality measured in the ICU, hospital and after 28 days of study.

Circulating IL-3 levels were assessed in relation to hospital mortality (primary outcome) and the following: 28-day mortality; ICU mortality; occurrence of septic shock; occurrence of specific acute organ dysfunction; positive blood cultures; and lengths of hospital and ICU stay.

Blood samples were obtained at time of inclusion and the third and seventh day of follow-up. The serum samples were obtained from blood collected for routine ICU analyses. The serum was stored in a -80ºC freezer until analysis. The serum IL-3 measurements were assayed all at once following completion of the study using the Human IL-3 DuoSet ELISA kit (R&D Systems, Minneapolis, MN, USA), according to the instructions provided by the manufacturer. The patient's pro- and anti-inflammatory cytokine profiles were also measured in samples collected at the time of inclusion. The Types 1, 2 and 17 T helper (Th1, Th2 and Th17) response cytokine profiles (IL-2, IL-4, IL-6, IL-10, tumor necrosis factor - TNF, Interferon-γ - IFN, and IL-17) were measured using the Cytometric Bead Array (CBA) method, following the instructions supplied by the manufacturer (BD Bioscience, San Diego, CA, USA).

### Statistical analysis

The sample size determination is described in the Supplementary material. Categorical variables are presented in terms of their absolute and relative frequency. Averages and standard deviations are used to describe continuous variables of normal distribution; and medians and interquartile ranges for continuous variables of nonnormal distribution. Comparisons between categorical variables were achieved through the chi-squared test or Fisher's exact test. Continuous variables were compared using Student's t-test or Mann-Whitney U test. Correlations between continuous variables were made using the Spearman correlation coefficient. Receiver operating characteristic (ROC) curves were plotted to establish the accuracy of the molecules analyzed in predicting the evaluated outcomes. Prognostic factors influencing the studied outcomes were initially assessed using univariate analysis models, and those with a p-value lower than 10% were assessed using multivariate analysis. Logistic regression models were applied for categorical outcomes, linear regression for continuous variables and Cox proportional risk analysis for time-dependent variables. Prediction models were compared using outcome measures (interclass correlation, coefficient of determination and Akaike Information Criteria - AIC).

Bicaudal test and a p significance value of 0.05 were defined for all analyses. The data was analyzed using Statistical Package for the Social Sciences (SPSS) version 20.1 (SPSS, Chicago, IL) and R version 3.1.1 (R Foundation for Statistical Computing, Vienna, Austria) software.

## RESULTS

**Figure 1:**
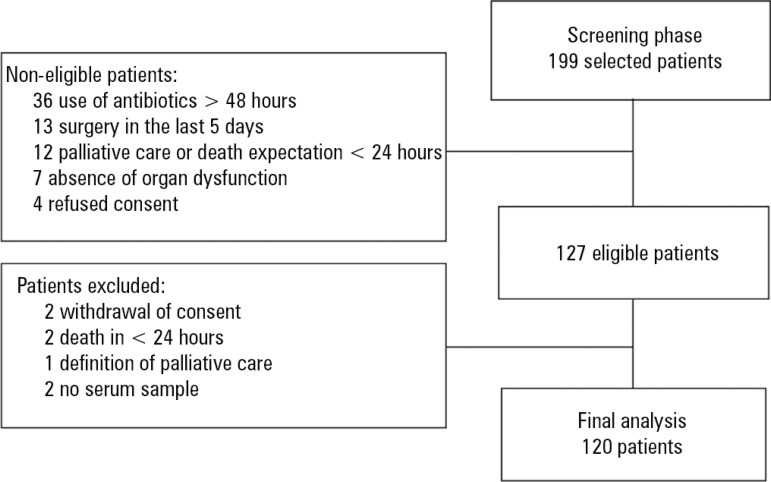
Patient flow diagram.

A total of 199 patients were screened for potential eligibility. Of this total, 127 (63.8%) patients were considered eligible, from whom 7 were excluded. Therefore, 120 patients were included in the final analysis (Figure 1).

The main characteristics of the patients included in the study are described in table 1. The 28-day, ICU and hospital mortality rates were 24%, 22.5% and 34%, respectively. The average age of the population was 55 years (± 18 SD) but was higher in the deceased patients. The most frequent comorbidities were arterial hypertension, diabetes mellitus and solid neoplasia. Among the assessed comorbidities, only nondialytic chronic kidney disease was significantly correlated with the primary outcome, *i.e.*, hospital mortality. APACHE II and SOFA score median values at inclusion were significantly higher in deceased patients when compared with survivors during hospital stay (Table 1).

**Table 1:** Patient characteristics at admission as a function of in-hospital mortality

Characteristics	Overall (n = 120)	Survivors (n = 79)	Decedents (n = 41)	p value
Age (years)	55 ± 18	51 ± 17	64 ± 14	< 0.001
Sex				0.1
Males	68 (57)	41 (52)	27 (66)	
Females	52 (43)	38 (48)	14 (34)	
Comorbidities				
Systolic heart failure	16 (13)	8 (10)	8 (20)	0.14
Solid malignancy	26 (22)	15 (20)	11 (27)	0.37
Hematologic malignancy	2 (1.7)	2 (2)	0 (0)	0.30
COPD	5 (4)	5 (7)	0 (0)	0.09
Liver cirrhosis	6 (5)	3 (4)	3 (8)	0.39
Chronic kidney disease	11 (9)	4 (5)	7 (18)	0.027
Dialytic chronic kidney disease	5 (4)	4 (5)	1 (2)	0.49
Arterial hypertension	51 (42)	29 (38)	22 (56)	0.06
Diabetes mellitus	24 (20)	13 (17)	11 (27)	0.18
Previous use of corticosteroids	6 (5)	5 (6)	1 (2)	0.34
Type of admission				0.29
Clinical	89 (74)	61 (77)	28 (68)	
Surgical	31 (26)	18 (23)	13 (32)	
APACHE II	17.5 (12 – 22)	15 (12 - 19)	21 (17 - 27)	< 0.001
SOFA	8 (6 - 11)	7 (5 - 9)	10 (8 - 13)	< 0.001

COPD - Chronic Obstructive Pulmonary Disease; APACHE II - Acute Physiology and Chronic Health Evaluation II; SOFA - Sequential Organ Failure Assessment. The results are expressed as the mean ± standard deviation, n (%) or median Q1 - Q3.

### Sepsis episodes and prognosis

Data regarding the aspects of sepsis episodes and its evolution are described in table 2. The majority of patients presented with septic shock (83%). Microbiological confirmation of sepsis was obtained for 75 (63%) patients, of which 51 (42.5%) had positive blood cultures. Occurrences of septic shock, mechanical ventilation, renal replacement therapy and use of inotropes were significantly correlated with death during hospital stay. Among the laboratory tests assessed in clinical routine, lactate and CRP levels at inclusion were also associated with hospital mortality (Table 2).

**Table 2:** Clinical and laboratory characteristics of sepsis episodes as a function of in-hospital mortality

Characteristics	Overall (n = 120)	Survivors (n = 79)	Decedents (n = 41)	p value
Type of infection				0.97
Community	32 (27)	21 (27)	11 (27)	
Nosocomial	88 (73)	58 (73)	30 (73)	
Confirmed microbiology	75 (63)	47 (60)	28 (68)	0.34
Positive blood culture	51 (43)	32 (41)	19 (46)	0.54
Microbiological documentation				0.20
Gram-positive bacteria	19 (16)	11 (14)	8 (19.5)	
Gram-negative bacteria	45 (38)	31 (39)	14 (34)	
Focus of infection				0.45
Lung	40 (33)			
Abdomen	29 (24)			
Urinary tract	9 (7)			
Catheter	13 (11)			
Skin and soft tissues	10 (8)			
Central nervous system	1 (1)			
Others	18 (15)			
Sepsis severity				0.01
Severe sepsis	20 (17)	18 (23)	2 (5)	
Septic shock	100 (83)	61 (77)	39 (95)	
Length of stay in ICU (days)	12 (4 - 21)	10 (4 - 18)	17 (8 - 28)	0.033
Length of stay in hospital (days)	37 (21 - 63)	46 (26 - 73)	34 (21 - 52)	0.075
Need for mechanical ventilator	91 (76)	51 (65)	40 (98)	< 0.001
Need for renal replacement therapy	34 (29)	12 (15)	22 (54)	< 0.001
Inotropes first 72 hours	22 (18)	10 (13)	12 (29)	0.026
Steroids first 72 hours	39 (33)	21 (27)	18 (44)	0.055
Leucocytes D1 g/L	15.6 (10 - 21)	15,6 (9.7 - 21)	17.8 (10.8 - 21)	0.61
Lactate D1 mg/dL	2 (1.2 - 3)	1,7 (1.2 - 2.8)	2.55 (1.7 - 3.5)	0.006
CRP D1 mg/L	237 (177 - 338)	220 (166 - 323)	256 (208 - 357)	0.042
Urea D1 mg/dL	65 (35 - 89)	48 (30 - 80)	87 (56 - 118)	< 0.001
PT D1	1.3 (1.1 - 1.6)	1.27 (1.1 - 1.5)	1.41 (1.2 - 2)	0.006

ICU - intensive care unit; CRP - C-reactive protein; D1 - Day 1; PT - prothrombin time. The results are expressed as n (%) or median Q1 - Q3.

### IL-3 and primary outcome

**Figure 2:**
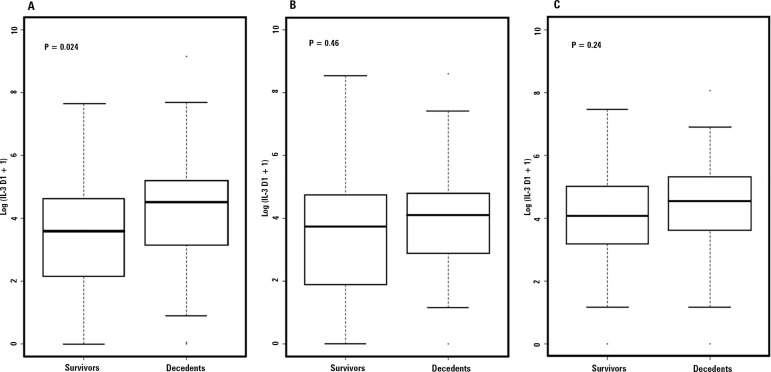
Circulating levels of IL-3 on days 1, 3 and 7 according to in-hospital survival. Box plots with median levels of interleukin-3 (IL-3), interquartile ranges and 10^th^ and 90^th^ percentiles on days 1 (A), 3 (B), and 7 (C). Levels of IL-3 expressed in logarithmic form.

**Figure 3:**
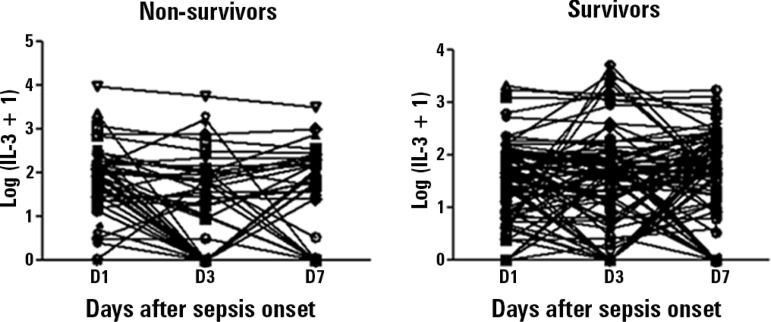
Sequential behavior of IL-3 levels on days 1, 3 and 7, according to in-hospital survival. IL-3 - Interleukin-3; D1 - Day 1; D3 - Day 3; D7 - Day 7. Levels of IL-3 expressed in logarithmic form.

Serum IL-3 levels were measured at the time of inclusion (day 1) in serum samples of all patients included in the study, on day 3 in 110 (92%) patients and on day 7 in 103 (86%) patients (Table 3). Median IL-3 serum levels measured upon inclusion were revealed to be statistically significantly higher in patients who died than in patients who survived the hospital stay, with values of 91.2pg/mL (21.7 - 182.6pg/mL) *versus* 36pg/mL (7.0 - 101.8pg/mL), p = 0.024, as shown in table 3. Hospital mortality was not associated with IL-3 levels measured on days 3 and 7 (Figure 2 and Figure 1S - Supplementary material). IL-3 had a markedly erratic behavior during sepsis episodes (Figure 3) , and no statistically significant differences were found between downward trend in the values of the biomarker and in-hospital survival (p = 0.185 for D1 - D3 trend and p = 0.169 for D1 - D7 trend).

**Table 3:** Serum cytokine levels in septic patients as a function of in-hospital mortality

Cytokines	Overall (n = 120)	Survivors (n = 79)	Decedents (n = 41)	p value
IL-3 D1 pg/mL	48.3 (11.8 - 127.4)	36 (7 - 101.8)	91.2 (21.7 - 182.6)	0.024
IL-3 D3 pg/mL	45.6 (10.5 - 114.7)	41.2 (9 - 113.7)	59.4 (14.6 - 127)	0.46
IL-3 D7 pg/mL	62.4 (23.2 - 167)	58.2 (16.4 - 162)	95 (37.2 - 201.7)	0.24
IL-2 D1 pg/mL	9.4 (8.2 - 11.2)	9.43 (8.4 - 11.3)	9.7 (8.1 - 11.1)	0.97
IL-4 D1 pg/mL	2.9 (2.5 - 3.3)	2.93 (2.4 - 3.3)	3 (2.5 - 3.3)	0.98
IL-6 D1 pg/mL	525.4 (118.5 - 5101.8)	402 (57.2 - 2704.5)	2083 (359 - 18608)	0.001
IL-10 D1 pg/mL	4.9 (2.9 - 10.9)	4.4 (2.7 - 8.3)	6.6 (3.7 - 31.2)	0.008
TNF D1 pg/mL	3.8 (3 - 4.6)	3.74 (3 - 4.5)	4.1 (3.3 - 4.7)	0.14
INF D1 pg/mL	4.7 (3.9 - 6.1)	4.9 (4 - 6.4)	4.7 (3.8 - 5.9)	0.45
IL-17 D1 pg/mL	11.6 (7.7 - 16.8)	11.6 (7.7 - 15.4)	10.5 (7.7 - 20.9)	0.76

IL- interleukin; D1 - Day 1; D3 - Day 3; D7 - Day 7; TNF - tumor necrosis factor; IL-17 - interleukin-17; INF - gamma interferon. The results are expressed as median Q1 - Q3.

In a Cox survival model, using hospital mortality as a dependent variable, IL-3 values measured at inclusion remained independently associated with prognosis, after adjusting for patient's age and sequential SOFA values on days 1, 3 and 7 (HR 1.032 95%CI: 1.010 - 1.055; p = 0.005) (Table 1S - Supplementary material).

**Figure 4:**
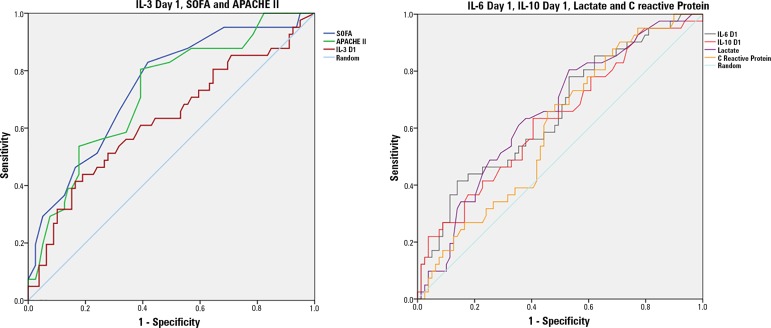
ROC curve of biomarkers and clinical scores. Outcome: All-cause in-hospital mortality. IL - interleukin; SOFA - Sepsis Organ Failure Assessment; APACHE - Acute Physiology and Chronic Health Evaluation; D1 - day 1.

To further investigate the accuracy of IL-3 to predict the outcome of septic patients, we built an ROC curve and found an area under the ROC curve of 0.62 (95%CI 0.51 - 0.73, p = 0.024) for hospital mortality (Figure 4). A cutoff initial IL-3 value above 127.5pg/mL was associated with hospital mortality (OR 2.97, 95%CI: 1.27 - 6.97; p = 0.0019), but with a low performance, as follows: 82% for specificity, 39% for sensibility, 53% for the positive predictive value, 72% for the negative predictive value, 0.73 for the negative likelihood ratio and 2.16 for the positive likelihood ratio, leading to a small variation in the posttest compared with the pretest death probability.

Despite the low accuracy of IL-3 to predict outcome in sepsis, we further evaluated if IL-3 might add to the prognostic information provided by the SOFA score. To this end, we carried out performance measures of hospital mortality prediction models constructed with and without IL-3. Interclass correlation, coefficient of determination (R^2^) and AIC measures were employed. Comparing the model constructed with sequential SOFA values and age with the model that made use of these same variables plus IL-3 levels on day 1, the three criteria applied agreed that the model that included IL-3 values was superior (Table 2S - Supplementary material), but with small variations that lack additional clinical significance.

### IL-3 and secondary outcomes

No difference was found in IL-3 levels at inclusion between patients with sepsis and septic shock. In addition, IL-3 levels were neither associated with specific organ dysfunctions - including septic shock - nor with ICU and 28-day mortality. Moreover, serum IL-3 levels measured on day 1, 3 or 7 were not shown to be associated with positive blood culture. However, among patients with positive blood culture (n = 51), IL-3 levels measured at the time of inclusion were shown to be statistically significantly higher among patients who died in intensive care (p = 0.007) and during the hospital stay (p = 0.024). No statistically significant difference was found in IL-3 levels between patients receiving or not receiving corticosteroids in the first 72 hours. Correlations between IL-3 levels and duration of ICU or hospital stay were not found, nor were they found with the severity scores and other biomarkers.

### Other cytokines and outcomes

The IL-2, IL-4, IL-6, IL-10, TNF, IFN and IL-17 cytokine levels were measured only upon inclusion (Table 3). IL-6 and IL-10 cytokine levels were shown to be associated with sepsis prognosis, as they were higher in the group of patients who died during hospital stay. In a correlation analysis, IL-6 and IL-10 levels at time of inclusion were strongly correlated with each other, with r = 0.772, p < 0.001. These cytokines were also positively correlated with SOFA scores at the time of inclusion, with r = 0.359 (p < 0.001) and 0.375 (p < 0.001), respectively. No correlation was found between the levels of these markers and other studied cytokines (including IL-3). Finally, IL-3 added no additional prognostic value to the other cytokines tested in this study (data not shown).

## DISCUSSION

In this study, serum IL-3 concentration was independently associated with all-cause hospital mortality in sepsis patients admitted to intensive care. However, the accuracy of IL-3 to predict this outcome proved to be low, which makes IL-3 a marker of little use in clinical practice. IL-3 appeared recently as a plausible prognostic marker in sepsis. Using a mouse model, Weber et al.^(9)^ elegantly demonstrated a key role of this molecule in sepsis pathophysiology, notably through the induction of myelopoiesis of Ly-6C monocytes and neutrophils and enhancement of cytokine levels. The authors further tested the prognostic role of IL-3 in two small cohorts of humans with sepsis and found that high plasma IL-3 levels were associated with high mortality even after adjusting for disease severity.

Despite its statistical association with prognosis in sepsis - likewise the above-mentioned study - the performance of IL-3 found here was not superior to that of previously studied biomarkers^(16-21)^ and clinical scores.^(22,23)^ The performance of IL-3 demonstrated through the area under the ROC curve was weak, as well as that presented by other biomarkers traditionally studied, revealing the difficulty in obtaining relevant prognostic information on sepsis with the use of isolated markers or clinical data.

The differences between the results obtained by this and the study of Weber et al. may be explained by differing patients' characteristics. Weber et al. assessed a population of 97 patients originating from two distinct cohorts, one of which was retrospective. The patients were, on average, older (average 65.8 ± 13.6 years) and apparently more severely ill (higher severity score and mortality) compared with those included in the present study. Additionally, the initial measurement of IL-3 in the study by Weber et al. was done in the first 24 hours of sepsis evolution, in contrast with the 48-hour window adopted in our study.

In addition, in their study, despite testing the independent association between IL-3 plasma levels and sepsis mortality, Weber et al. did not explore the accuracy of this marker to predict this outcome.^(9)^ Other factors that potentially explain the differences found in both studies relate to the genetic differences between the populations studied.^(24,25)^

We anticipated that the behavior of serum IL-3 levels over the course of sepsis management could be useful to evaluate the response to antibiotic therapy. However, in contrast to inflammatory markers such as CRP^(26)^ and procalcitonin,^(27,28)^ IL-3 had a markedly erratic behavior. In addition, the weak or absent synchrony of IL-3 levels with the levels of other classically studied biomarkers, as well as with the severity scores values, and the absence of associations between IL-3 levels and the secondary outcomes, indicate the need for further investigations of the behavior and utility of this cytokine in sepsis patients.

Even though the analysis of IL-3 values failed to show any particular usefulness in this case, the individualized approach with immunologic and inflammatory status characterization of patients through clinical data and biomarker and cytokine panels may provide a better management of sepsis.^(29-31)^ As an example of this type of approach, two meta-analyses revealed a putative beneficial use of corticoids in patients admitted for community-acquired pneumonia (CAP).^(32,33)^ Nonetheless, evidence suggests that the group of patients with severe CAP associated with exacerbated inflammatory response (characterized by higher levels of CRP) would show the best response to this therapy.^(34)^ In this scenario, IL-3 dosing, added to a panel of biomarkers, performed using a simple and reproducible technique, could assist in identifying a subgroup of patients with worse prognosis who would be, for instance, potential candidates for anti-inflammatory therapy. However, for this purpose, additional studies are needed.

Regarding the other cytokines assessed in this study, IL-6 and IL-10 levels were revealed to be significantly elevated in patients who died during hospital stay, in agreement with previously published data,^(5,19,35-38)^ suggesting that both enhanced inflammation and sepsis-related immunosuppression are defining factors of bad prognosis. Lastly, contrary to what was expected considering previously published data,^(9)^ in our study, we did not find any correlations between IL-3 concentration and other inflammatory cytokine levels or with leukocyte and neutrophil counts.

Our study holds numerous limitations. It was a study carried out in a single center, with a relatively small patient sample, which limits the power of statistical analysis and the extrapolation of our findings. There was no control group for IL-3 or other cytokine dosage in healthy or sepsis-free critically ill patients. However, as cytokine measurements are already well standardized and the study's primary objective was to evaluate the impact of the levels of these molecules in clinical outcomes in a specific population, this dosage was not mandatory. The time of serum sample collection might also have been a limiting factor, given the 48-hour window for patient inclusion in the study. Beyond this time window leading to a possible heterogeneity of the sample, measurements taken in later serum collection might not reliably reflect IL-3 levels during the start of the inflammatory cascade due to the short half-life of this biomarker.^(39)^

## CONCLUSION

In this study, with 120 septic patients in an intensive care unit, elevated IL-3 serum levels were independently associated with hospital mortality, but with poor clinical performance. The isolated use of this cytokine was not superior to other biomarkers and clinical scores classically used as predictors of outcome in sepsis. The benefit of using IL-3 as an isolated marker or as part of a biomarker panel for prognostic characterization and risk stratification in sepsis patients must be evaluated in future investigations.
