# Supplementary material for: Role of interleukin-3 as a prognostic marker in septic patients
Source: Rev Bras Ter Intensiva. 2018 Oct-Dec;30(4):443–52. doi: 10.5935/0103-507X.20180064 (PMC6334479; doi:10.5935/0103-507X.20180064)
Supplement: Supplementary file 1 [file rbti-30-04-0443-suppl1.pdf]

Role of interleukin-3 as a prognostic marker in septic patients

Avaliação da interleucina 3 como marcador prognóstico na sepse

Isabela Nascimento Borges[1], Carolina Braga Resende[1], Érica Leandro Marciano Vieira[2], José Luiz Padilha da Silva[3], Marcus Vinícius Melo de Andrade[4], Andrea Jerusa de Souza[1], Eurípedes Badaró[1], Rafael Mourão Carneiro[1], Antônio Lúcio Teixeira Jr.[1,2], Vandack Nobre[1] on behalf of Núcleo Interdisciplinar de Investigação em Medicina Intensiva (NIIMI)

Sample size determination

There is only one study published in the medical literature regarding the use of IL-3 as a prognostic marker in sepsis.[9] In that study, analysis of IL-3 levels in 97 patients (retrospective data of a previously published prospective cohort with 60 patients and prospective data of a new cohort with 37 patients) revealed that IL-3 was associated with 28-day follow-up mortality in severe sepsis or septic shock patients. Considering a cut-off value of 89.4pg/mL, 38% of deceased patients had IL-3 levels above that value, compared with 11% of the surviving patients, with a 4.9 odds ratio in the Kaplan-Meier survival curve. Global 28-day mortality was 37.1%. In the retrospective phase of the present study, we observed a 27.8% 28-day mortality rate. Considering the prevalence of positive IL-3 (> 89.4pg/mL) in surviving and deceased patients up to the 28th day of the aforementioned study and the allocation ratio of 0.38 in both patient subgroups in our retrospective cohort (i.e., 1 death for every 2.6 survivors), 95 survivors and 36 deaths would be required for a statistical significance to be found between the two groups. Because the primary outcome analyzed in our study was hospital mortality (as opposed to 28-day mortality), the measurement was adjusted considering a 32.9% hospital mortality rate in the first phase of our cohort. Thus, 40 deaths and 81 survivors during hospital stay (yielding a total of 121) would be required for a statistically significant difference to be found between these two subgroups, with a 95% power and 5% α-error.

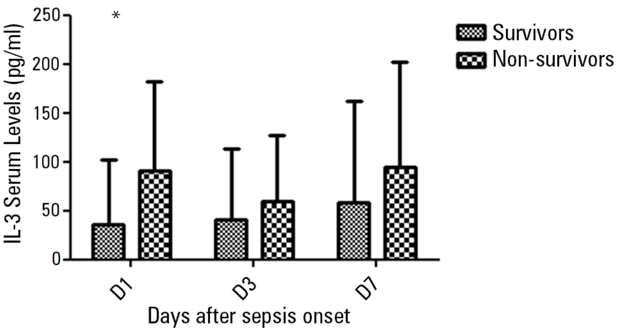

Figure 1S - Circulating levels of IL-3 on days 1, 3 and 7 according to in-hospital survival. Confidence interval bars with median levels of interleukin-3 (IL-3) and 75th percentiles on days 1 (D1), 3 (D3), and 7 (D7). * p statistically significant.

Table 1S - Hospital mortality adjusted for the covariables age and sequential SOFA - Cox survival analysis

Variable	Hazard ratio	95%CI	p value
IL-3 D1	1.032	1.010 - 1.055	0.005
SOFA D1, D3, D7	1.154	1.068 - 1.248	< 0.001
Age	1.035	1.010 - 1.060	0.5

95%CI - 95% confidence interval; IL-3 - Interleukin-3; D1 - Day 1; SOFA - Sequential Organ Failure Assessment; D3 - Day 3; D7 - Day 7.

Table 2S - Model performance measures - hospital mortality prediction

Model	Interclass correlation	Coefficient of determination (R^2)	AIC
IL-3 D1+ SOFA + AGE	0.777	0.063 (0.486)	234.20
SOFA + AGE	0.743	0.052 (0.483)	236.52

IL-3 - Interleukin-3; D1 - Day 1; SOFA - Sequential Organ Failure Assessment; AIC - Akaike Information Criteria.
